# Parallel Generative Topographic Mapping: An Efficient Approach for Big Data Handling

**DOI:** 10.1002/minf.202000009

**Published:** 2020-04-29

**Authors:** Arkadii Lin, Igor I. Baskin, Gilles Marcou, Dragos Horvath, Bernd Beck, Alexandre Varnek

**Affiliations:** ^1^ University of Strasbourg Laboratory of Chemoinformatics, Faculty of Chemistry 4, Blaise Pascal str. 67081 Strasbourg France; ^2^ Faculty of Physics Lomonosov Moscow State University 1/2, Leninskie Gory str. 119991 Moscow Russia; ^3^ Department of Medicinal Chemistry Boehringer Ingelheim Pharma GmbH & Co. KG 65, Birkendorfer str. 88397 Biberach an der Riss Germany

**Keywords:** Parallel Generative Topographic Mapping, Big Data, Frame set, ChEMBL

## Abstract

Generative Topographic Mapping (GTM) can be efficiently used to visualize, analyze and model large chemical data. The GTM manifold needs to span the chemical space deemed relevant for a given problem. Therefore, the Frame set (FS) of compounds used for the manifold construction must well cover a given chemical space. Intuitively, the FS size must raise with the size and diversity of the target library. At the same time, the GTM training can be very slow or even becomes technically impossible at FS sizes of the order of 10^5^ compounds – which is a very small number compared to today's commercially accessible compounds, and, especially, to the theoretically feasible molecules. In order to solve this problem, we propose a Parallel GTM algorithm based on the merging of “intermediate” manifolds constructed in parallel for different subsets of molecules. An ensemble of these subsets forms a FS for the “final” manifold. In order to assess the efficiency of the new algorithm, 80 GTMs were built on the FSs of different sizes ranging from 10 to 1.8 M compounds selected from the ChEMBL database. Each GTM was challenged to build classification models for up to 712 biological activities (depending on the FS size). With the novel parallel GTM procedure, we could thus cover the entire spectrum of possible FS sizes, whereas previous studies were forced to rely on the working hypothesis that FS sizes of few thousands of compounds are sufficient to describe the ChEMBL chemical space. In fact, this study formally proves this to be true: a FS containing only 5000 randomly picked compounds is sufficient to represent the entire ChEMBL collection (1.8 M molecules), in the sense that a further increase of FS compound numbers has no benefice impact on the predictive propensity of the above‐mentioned 712 activity classification models. Parallel GTM may, however, be required to generate maps based on very large FS, that might improve chemical space cartography of big commercial and virtual libraries, approaching billions of compounds

## Graphical abstract

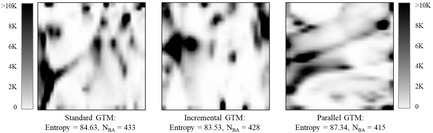

## 1 Introduction

Nowadays, public and private chemical databases contain millions of already synthesized compounds (ChEMBL,^[1]^ PubChem,^[2]^ CAS,^[3]^ etc.) and billions of computer‐generated virtual structures (GDB‐17^[4]^). This chemical universe needs to be explored and analyzed. Earlier, Oprea et al. proposed to use geography concepts to represent chemical structures on a map.^[5]^ Several methods designed to visualize and model chemical space are known in the literature: Scaffold‐Tree,^[6]^ PCA,^[7]^ Multi‐fusion similarity maps,^[8]^ t‐SNE,^[9]^ UMAP,^[10]^ TMAP,^[11]^ etc. Generative Topographic Mapping (GTM), introduced by Bishop et al.,^[12]^ has a particular advantage – it is a non‐linear probabilistic approach extending the Self‐Organizing Maps.^[13]^

The GTM algorithm considers a 2‐dimensional smooth surface (*manifold*) injected into the high‐dimensional descriptors space. The manifold is fitted to data distribution by maximizing the log‐likelihood (*LLh*) of the molecules in the input space defined by molecular descriptors. Once the manifold is fitted, the molecules are projected onto the 2‐dimensional latent space superposed with a square grid of k*k (*K*) nodes. To determine the position of each compound, a vector of posterior probabilities (*responsibilities*) to be associated with a given node is used. To describe the entire data set, a vector of cumulative responsibilities can be built using responsibility vectors of individual compounds. The latter can be associated with class or property values which leads to GTM Class Landscape or GTM Property Landscape. These landscapes can be used as classification and regression models in various chemoinformatics tasks.[^13^, ^14^, ^15^, ^16^, ^17^, ^18^, ^19^, ^20^, ^21^, ^22^, ^23^, ^24^, ^25^, ^26^, ^27^]

A map is built on a set of molecules called a Frame set (FS) that spans the given chemical space. Usually, the FS is taken as a small portion of compounds in comparison to the database size (10K to 30K molecules). The general expectation is that a larger FS should give a “better” map. However, it is not *a priori* clear how large the FS needs to be. Note that the FS is *not*, as one might expect, meant to be a representative “core” of a library, *i.e*. represent one non‐redundant example of every analogue contained by the parent library. By analogy to cartography, one should think of the FS as the “satellites” sufficient to ensure a desired resolution of the GPS location system. Most of the mapped molecules do not need to be by any means close analogues to the FS – yet, they have to be “surrounded” by several of FS members, in order to ensure the precise “triangulation” and projection on the map. As such, not only the number of FS compounds is of paramount importance, but also their homogeneous spread over chemical space (a few tens of satellites is sufficient to support GPS location within 10 m anywhere on Earth, but this would no longer be the case if all of them would be hovering over the same spot of the Atlantic). In previous works, FS selection was never thoroughly studied, since maps of good quality were typically obtained on hand of randomized compound subsets chosen as large as computational time and memory constraints would allow. A thorough analysis of this problem was hence due, and will be pursued in this work, all while introducing an original parallel GTM (pGTM) algorithm to cope with FS sizes not envisageable with previous map building tools.

The standard GTM (*sGTM*) algorithm is limited in terms of the size of the FS. This limitation rises when the machine needs to compute Euclidean distances for each pair “node‐molecule”. For instance, computing the distances between 900 nodes (a map of 30×30 nodes) and 1000 compounds described with some 500 descriptors takes approximately 4.5 seconds on a single CPU (Intel Core i7‐6700HQ). The complexity of the method is O(n). Hence, 30 K compounds already need 135 seconds or 2.25 minutes. An additional variation of the number of nodes makes the complexity to be O(n, k), and 2 K compounds in a pair with 1800 nodes already require 19 seconds to be treated. This procedure is performed at each iteration, which makes the GTM algorithm slow. This is acceptable for relatively small FSs (up to 30 K compounds), whereas it is better to use incremental GTM for larger ones.[^12^, ^22^] Within this algorithm, a data set is split into a number of blocks that are treated sequentially. The acceleration of the method is achieved due to the ability of the algorithm to converge faster on a sequence of blocks than on the entire data set. Such an approach, however, displays several drawbacks. First, it is faster than sGTM but, still, too slow because the convergence must be achieved on each block. Second, the manifold is initialized only with the first block chosen randomly, and then it is updated by the following blocks. The order of the blocks in the sequence impacts significantly the resulting manifold since the knowledge extracted from the middle blocks can fully or partially be lost at the end of the training procedure. Thus, for instance, the reshuffling of the training data set leads to a completely different GTM.

These problems become even more crucial in the case of Big Data. To accelerate the training procedure, the FS was necessarily limited to a subset of such big chemical libraries (*e. g*., more than 100 K compounds). Thus, a question on the optimal FS size arises. In the previous studies,[^18^, ^27^, ^28^] the size of the FS was either optimized by the Genetic Algorithm^[29]^ (GA) as one of the hyper‐parameters of the GTM model or specified manually based on the researcher's experience.^[17]^ Intuitively, one can assume that a larger chemical collection may need a larger FS to represent a given chemical space, whereas the GA was often selecting FSs of few thousands (5 K–25 K structures). This can be explained by assuming that the considered FS of the order of 10^3^–10^4^ randomly selected compounds effectively represents a huge chemical collection, such as ChEMBL (10^6^ compounds). Apparently, FS sizes of <1 % of the final targeted compound collection may – fortunately – be sufficient, but no rigorous study of the FS size has been conducted so far.

To overcome the FS size limitations all while rendering the manifold independent on the order of FS data blocks, we have developed a new parallel GTM (pGTM) algorithm. It was applied to investigate the optimal size of the FS suitable for producing a meaningful map for a large chemical collection, such as the ChEMBL database. In particular, we investigated whether increasing the FS size far beyond the so‐far employed 10^3^–10^4^ randomly selected compounds would significantly enhance the map quality using the pGTM approach. Different FSs ranging from 10 to 1.8 M compounds were prepared. Their representativity of the entire ChEMBL database was calculated in the initial descriptor space, using the Kullback‐Leibler divergence criterion. The maps were trained by pGTM, sGTM and iGTM algorithms, in as far as FSs size allowed it (sGTM and iGTM on smaller FS, iGTM/pGTM on larger ones, only sGTM and respectively only pGTM for extremely small and respectively large sets). The maps were analyzed from two points of view: (1) the homogeneity of the mapped compound density (Shannon Entropy) and (2) their predictive power in class landscape‐based polypharmacological activity prediction, as will be detailed in the Methods section.

## 2 Data and Descriptors

As a data source, the public chemical database ChEMBL (v.25) was used in this study. Chemical structures were preprocessed in 7 steps: dearomatization, removal of the explicit hydrogens, removal of the information on isotopes and stereo, stripping salts, aromatization, selection of a major tautomer, and transformation of common functional groups (e. g. nitro group).

The compounds possessing less than 5 or more than 100 heavy atoms were discarded. The obtained collection of about 1.8 M compounds was used to prepare 80 FSs of different sizes (10, 50, 100, 500, 1 K, 5 K, 10 K, 20 K, 30 K, 50 K, 100 K, 200 K, 400 K, 750 K, 1 M, and 1.8 M compounds) with five randomly selected FSs per each size (in the case of 1.8 M compounds, the FS was just reshuffled 5 times). The molecules were encoded by the 10,898 ISIDA fragment descriptors using the IA‐FF‐FS‐AP‐2‐3 fragmentation scheme (sequences of 2–3 atoms colored by CVFF^[30]^ and formal charges).[^27^, ^31^, ^32^] The near‐constant descriptor elements were removed (if the standard deviation was zero, or less than 2 % of the covered range width in the Frame set) and standardized (centered and divided by its standard deviation). Depending on the FS size, the final number of descriptors varied from 180 to 540.

To discard the compounds that are poorly described by the manifold (i. e., with large distances to the manifold), a Gaussian‐based GTM Applicability Domain (AD)^[17]^ was employed. Within this AD, a Gaussian is fitted to the LLh distribution built by binning the corresponding FS. The LLh threshold is computed as LLh_peak_−3σ, where LLh_peak_ is the LLh value corresponding to the peak of the fitted Gaussian, and σ is its width. Once the threshold is computed, compounds possessing the LLh below the threshold are discarded.

**Figure 1:**
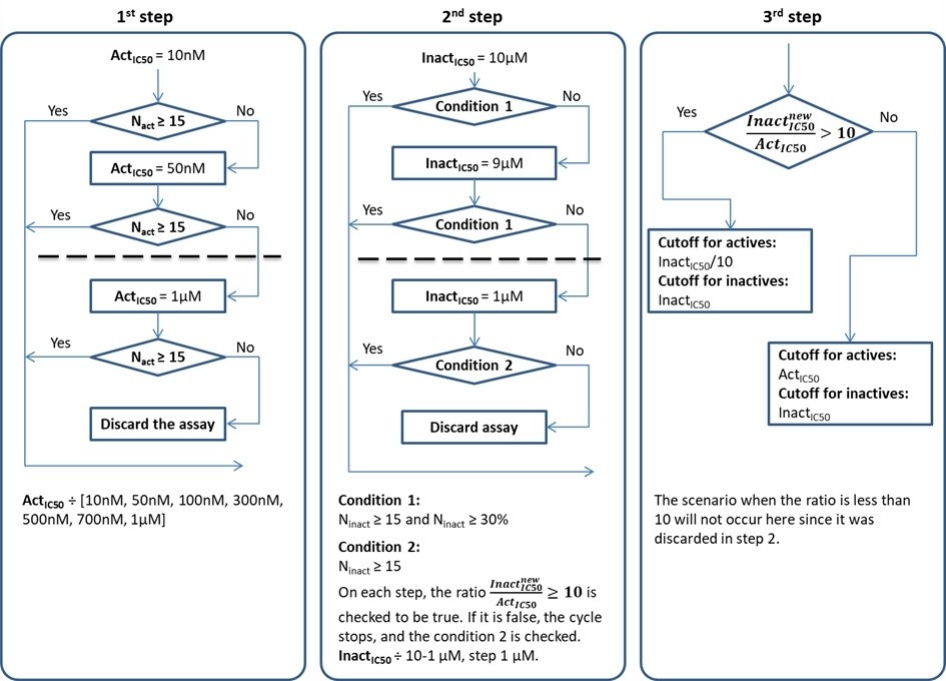
Labels assignment protocol. Here, three stages are depicted: 1) assessing of the preliminary IC50 threshold for actives (Act_IC50_); 2) determining of the IC50 threshold for inactive compounds (Inact_IC50_); 3) updating the Act_IC50_ as Inact_IC50_/10 if Inact_IC50_/Act_IC50_>10. The compounds with Act_IC50_<IC50<Inact_IC50_ are discarded.

To validate the maps, more than 1000 ChEMBL targets for “Homo sapiens” organism with assay type “Binding assay” and target type “Single protein” were preselected. They were filtered according to the number of compounds for which the IC50 value was measured (at least 30). The labels “active”/”inactive” were assigned based on the IC50 value according to the protocol depicted in Figure 1. Briefly speaking, the protocol defines the IC50 thresholds for each target individually in accordance with the number of active and inactive compounds. The protocol consists of three steps. First, the “active” IC50 threshold (Act_IC50_) is selected out of the range [10 nM, 50 nM, 100 nM, 300 nM, 500 nM, 700 nM, 1 μM] to define at least 15 actives. Next, it selects the “inactive” IC50 threshold (Inact_IC50_) which determines 30 % or at least 15 compounds as inactive. In the meantime, the condition “Inact_IC50_/Act_IC50_≥10” is checked. Finally, if the threshold for inactive compounds Inact_IC50_ is more than 10 times bigger than the threshold for actives (Act_IC50_), the Act_IC50_ is updated as Inact_IC50_/10 to collect more active molecules.

The targets with less than 15 active or 15 inactive compounds were discarded. The final number of the targets considered in the study varied from 0 to 712 depending on the size of the corresponding FS.

## 3 Method

### 3.1 Standard GTM

**Figure 2:**
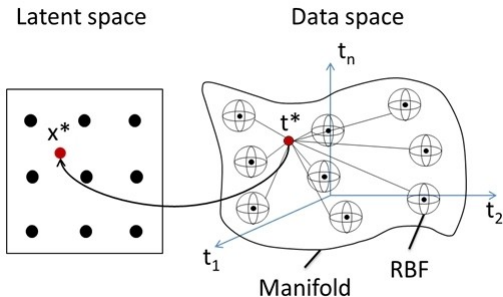
The basic idea of the GTM. Here, the data point t* from the multi‐dimensional data space (right) is projected to x* the 2D latent space (left) using the manifold which is injected into the data space and described by a set of Radial Basis Functions (RBF).

GTM is a probabilistic extension of the Self‐Organizing Mapping (SOM)^[33]^ method where log‐likelihood is utilized as an objective function.^[12]^ The manifold used to bind a data point t* in the data space and its projection x* in the latent space (Figure 2) is described by a set of *M* Radial Basis Function (*RBF*; Gaussian functions are used in the current implementation) centers.

To initialize the manifold, the parameter matrix **W** containing the RBF positions in the data space is obtained from the Principal Component Analysis (PCA)^[34]^ performed for the descriptors matrix as

(1) $$W=\Phi^{-1}\left(XU\right).$$

Here, **Φ** is *K×M* matrix containing relative RBF positions in the latent space with respect to the nodes:

(2) $$\Phi_{\mathrm{km}}=\exp\left(\frac{∥x_{k}-\mu_{m}{∥}^{2}}{2\sigma^{2}}\right),$$

where x_k_ and μ_m_ are the coordinates of a node k and an RBF center m in the latent space, respectively, and σ is the average squared Euclidean distance between two RBF centers multiplied by a tunable factor *w*; **X** is *K*×2 matrix of nodes’ coordinates in the latent space (according to the square grid which represents the latent space), and **U** is 2×*D* matrix of the first two eigenvectors produced by PCA.

Once the manifold is initialized, the mapping function **Y** is computed as

(3) Y=ΦW,

which is *K*×*D* matrix projecting the nodes from the latent to the initial space. Next, the initial log likelihood value LLh(**W**, β) is computed using the 3^rd^ eigenvalue issued from PCA calculations at the manifold initialization step as an initial guess of β^−1^ (the noise variance)

(4) $$LLh\left(W,\beta \right)=\frac{1}{N}\sum_{n=1}^{N}\ln\left\{\frac{1}{K}\sum_{k=1}^{K}p\left(t_{n}|x_{k},W,\beta \right)\right\},$$

(5) $$p\left(t_{n}|x_{k},W,\beta \right)={\left(\frac{\beta }{2\pi }\right)}^{-D/2}exp\left(-\frac{\beta }{2}{∥y_{k}-t_{n}∥}^{2}\right),$$

where **t_n_** is the position of a molecule *n* in the data space, **y_k_** is the position of a node *k* in the data space (obtained via eq. 3). These conditional densities (eq. 5) are transformed then into posterior probabilities (*responsibilities*)

(6) $$r_{nk}=\frac{p\left(t_{n}|x_{k},W,\beta \right)}{\sum_{k^{{}^{'}}=1}^{K}p\left(t_{n}|x_{k^{{}^{'}}},W,\beta \right)},$$

and the Expectation‐Maximization (EM) algorithm is run to fit the manifold. Within the training procedure, the matrix **W** and the value of β are updated as

(7) $$W={\left(\Phi^{T}G\Phi +\lambda I\right)}^{-1}\Phi^{T}RT,$$

(8) $$\frac{1}{\beta }=\frac{1}{ND}\sum_{n=1}^{N}\sum_{k=1}^{K}r_{kn}{∥y_{k}-t_{n}∥}^{2}.$$

The EM algorithm maximizes the log‐likelihood of compounds to be described by the nodes and stops once the convergence is achieved. Finally, the projected data set is described by the *N*×*K* matrix of responsibilities **R** (eq. 6), or by a vector of cumulated responsibilities (sum of responsibilities at a given node over the entire data set) of length *K*.

### 3.2 Incremental GTM

To overcome the limitation of the sGTM described above (the number of training compounds), the Incremental GTM algorithm was introduced.^[12]^ Within this approach, a manifold is initialized by a randomly chosen subset. Next, the data set is split into a series of blocks of a certain size which are used to train the manifold sequentially. This solves the problem of the number of training compounds and allows treating of larger FSs. At the same time, the manifold is built slower since the convergence must be achieved on each block. In addition, the GTM training of the FS of more than 100 K–200 K compounds becomes too costly in terms of computational time which means that a new GTM method able to handle larger FSs in a relatively short time is needed.

### 3.3 Parallel GTM

An attempt to parallelize the sGTM algorithm was already made using Message Passing Interface (*MPI*) technique.[^35^, ^36^, ^37^] For this purpose, the matrix of responsibilities was decomposed and its parts were distributed over the CPUs to be updated by small chunks of the data set iteratively. This accelerated the manifold training, but the mentioned approach is dependent on the certain architecture of a machine used to run the calculations. Namely, a single machine or a highly organized cluster that supports the MPI technology must be used for calculations, and the RAM has to be shared between the machines to store the whole matrix of responsibilities.

**Figure 3:**
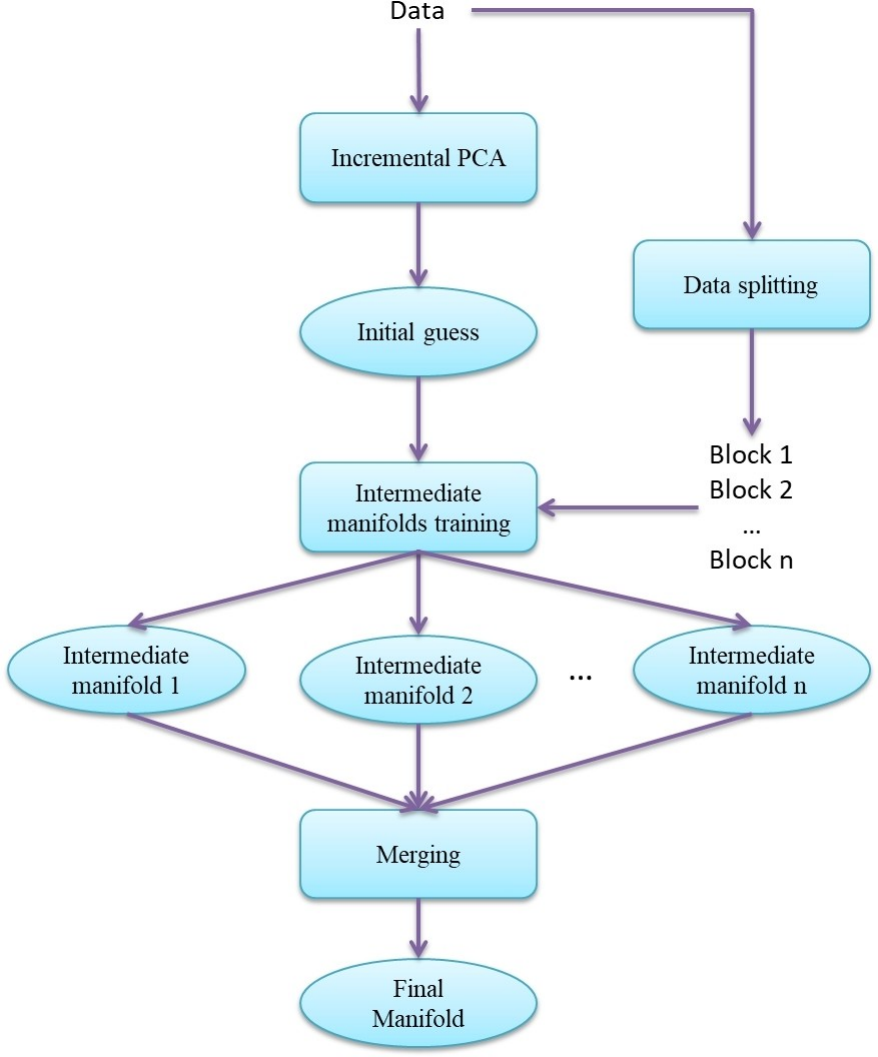
The Parallel GTM algorithm.

Here, we present a new solution named Parallel GTM (pGTM) which extends the iGTM to multiple CPUs. The idea is to generate, first, a common initial guess valid for the given FS, and then to distribute the tasks over the cluster in order to fit each data block independently. The workflow is presented in Figure 3.

Within this approach, the parameter matrix **W** is initialized on the entire FS using the incremental Principal Components Analysis (iPCA). To do so, the covariance matrix is computed incrementally followed by the Eigenvalue decomposition^[38]^ (the scikit‐cuda library in Python was applied).^[39]^ Once the PCA is done, the FS is split into a series of blocks, and, then, the manifold training procedure is executed to fit each block independently.

Each block produces an intermediate GTM manifold fitted on a portion of the data set. Since the same initial position of the manifold in the descriptors space and the same GTM parameters are used to treat the blocks, the intermediate manifolds can be then merged into the final one. For this purpose, simple averaging of the matrices **W** and the noise variances *β* can be used:

(9) $${\bar{w}}_{m,d}=\frac{\sum_{i=1}^{N}w_{m,d,i}}{N},$$

(10) $$\bar{\beta }=\frac{\sum_{i=1}^{N}\beta_{i}}{N},$$

where *N* is the number of data blocks used to train the manifold.

### 3.4 Benchmarking Strategy

Each of the 80 FSs was used to train a GTM with the GTM parameters taken from the previous study:^[12]^ 841 nodes, 324 RBFs, the regularization coefficient of 3.236, and the RBF's width of 0.4. For benchmarking purposes, the FSs were treated by three GTM algorithms: sGTM, iGTM, and pGTM. The standard approach was applied to the FSs of 10 to 30 K compounds. The incremental algorithm was applied to the FSs of 5 K–200 K compounds. The pGTM algorithm was used to build the maps on FSs containing 5 K–1.8 M compounds (smaller FSs were not used with iGTM and pGTM which are intrinsically less accurate technical “back‐up” solutions meant for processing FSs too large or too time‐consuming for sGTM). The FSs of sizes close to the applicability thresholds of the methods (corresponds to the FS sizes analyzed by the methods) were processed by several methods. The data blocks used for iGTM and pGTM contained no more than 5 K compounds.

The FSs representativity was checked, and the obtained maps were compared in terms of the homogeneity of the mapped compound density, and the polypharmacological predictive performance as defined below.

#### 3.4.1 Frame Set Representativity

Within a particular descriptor, an FS, as well as the chemical collection, can be represented as a probability distribution obtained by binning the corresponding standardized descriptor values. The probability distributions *p_i_(x)* obtained for the FS and *q_i_(x)* for the entire chemical collection can be then compared to assess the FS representativity within the *i*‐th descriptor. In an ideal case, *p_i_(x)* should fully mimic the *q_i_(x)*, and the Kullback‐Leibler divergence (*KLD_i_*),^[40]^ computed as:

(11) $${KLD}_{i}=\int p_{i}\left(x\right)\log\frac{p_{i}\left(x\right)}{q_{i}\left(x\right)}dx$$

should be equal to zero. In the case of a non‐representative FS, the *KLD_i_* tends to be infinite. To extend this to the multi‐dimensional distributions, the single‐dimension *KLD_i_* values were averaged, and the mean *KLD* and its standard deviation were computed.

#### 3.4.2 Compound Density Distribution on the Map (in the Latent Space)

To measure the uniformity of compound distribution, the normalized Shannon entropy can be used as a metric. For this purpose, the vector of cumulative responsibilities is created using the compounds passed the LLh filtering (the compounds with LLh lower than the threshold were discarded; the LLh threshold is explained in the chapter “2 Data and Descriptors”). The Shannon entropy is computed as

(12) $$E=-\sum_{k}{CumR}_{k}log\left({CumR}_{k}\right),$$

which can be normalized then dividing it by the maximal entropy *log(K)*:

(13) $$E_{norm}=\frac{-\sum_{k}{CumR}_{k}log\left({CumR}_{k}\right)}{log\left(K\right)}{}^{*}100,$$

Here, CumR_k_ is the cumulated responsibility in the node *k*, and K is the total number of nodes. The E_norm_ (normalized entropy) ranges within [0;100], where 0 means that all the molecules are mapped into the same node, and 100 means that the molecules cover the chemical space uniformly.

#### 3.4.3 Predictive Performance

Predictive performance is a key indicator of the relevance of a GTM manifold.^[14]^ This was estimated in terms of three‐fold cross‐validated classification challenges of active versus inactive compounds associated with a large profile of ChEMBL biological targets, following the “universal map” paradigm.[^18^, ^21^, ^28^] Within the cross‐validation procedure, a target‐specific data set was split into three folds, and a GTM class landscape (not a manifold) was trained on two folds and evaluated by the third one. *Balanced Accuracy* (BA) was applied in this study to assess the predictive performance of the maps. Upon projection of an item to be predicted on the activity‐specific two‐class classification manifold (1=inactive, 2=active), the returned real score indicates the predicted likelihood of the compound is a member of the class closest to the rounded‐up score. Therefore, to compute the BA, this score is simply rounded up to the next integer as a predictor of the most likely activity class. As a result, each target was characterized by the mean BA values. To compare the maps, the targets predicted with the <BA>≥0.7 were counted.

## 4 Results and Discussion

### 4.1 Comparison of Different Algorithms of GTM Construction for a Given Frame Set

**Figure 4:**
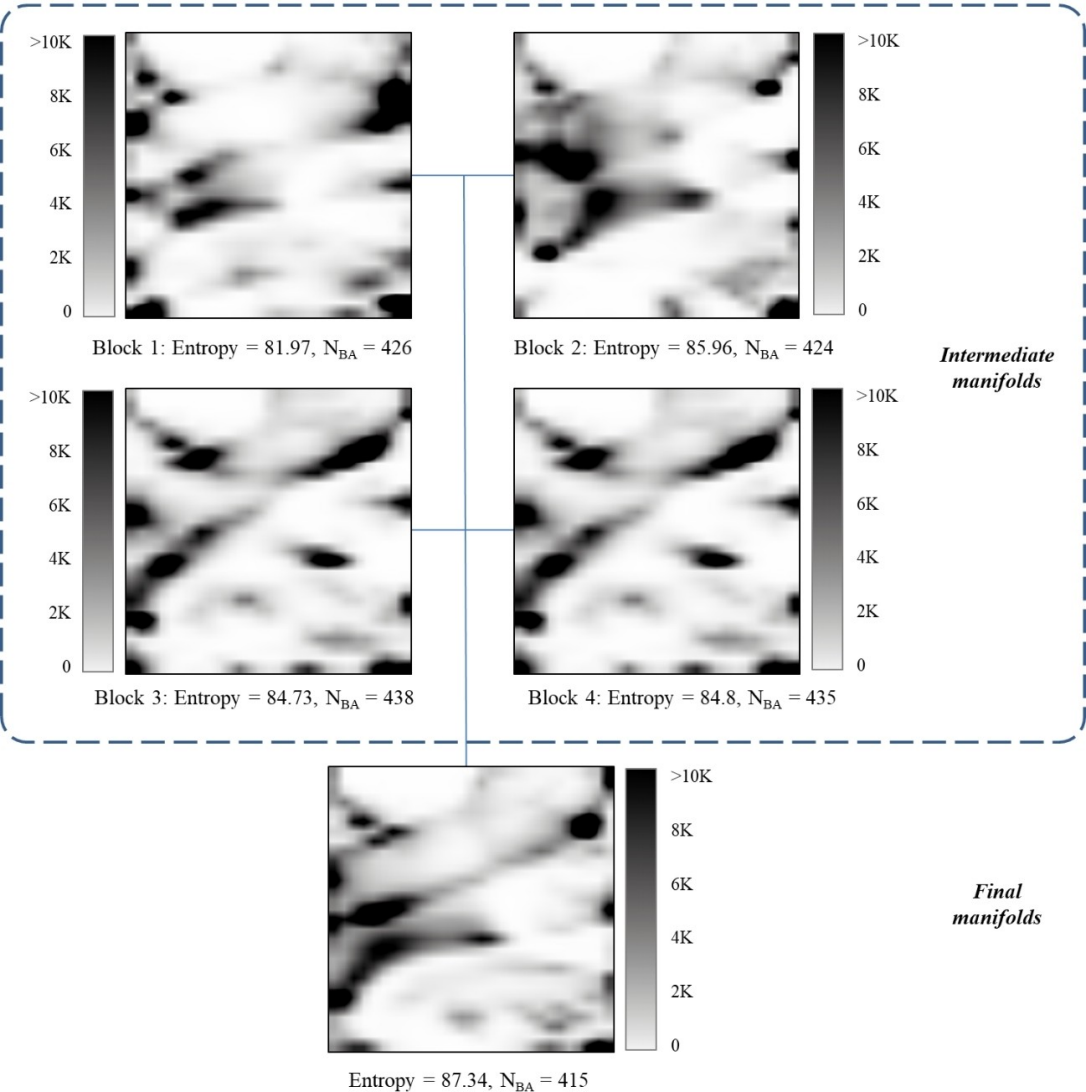
GTM density landscapes built for the intermediate and final pGTM manifolds. To build the landscapes, the entire ChEMBL collection was projected on each manifold. Here, an FS of 20 K compounds was split into four blocks to train the intermediate manifolds. Each manifold was described then by the normalized Shannon entropy (Entropy) and the number of targets predicted with <BA>≥0.7 (N_BA_).

The parallel GTM algorithm was tested, first, on an FS of 20 K compounds. For this purpose, four intermediate GTM manifolds were trained on 5 K compounds each, and the entire ChEMBL collection was projected on them as well as on the final manifold. The obtained projections were used to train GTM density landscapes, to compute the normalized Shannon entropy and to count the targets predicted with the <BA>≥0.7 (N_BA_; Figure 4).

Analysis of the produced landscapes shows that the intermediate manifolds are similar to each other visually, although they describe different parts of the FS.

The entropy of the intermediate manifolds is 84.3±1.7, and the N_BA_ is 431±7. Merging them into the final manifold, we increase the entropy (87.34) but decrease the predictive performance (415 targets were predicted with the <BA>≥0.7). Thus, the plain averaging of manifolds appears to be slightly detrimental on prediction quality – in perspective, alternative ways to merge local manifolds into the global one need to be addressed.

**Figure 5:**
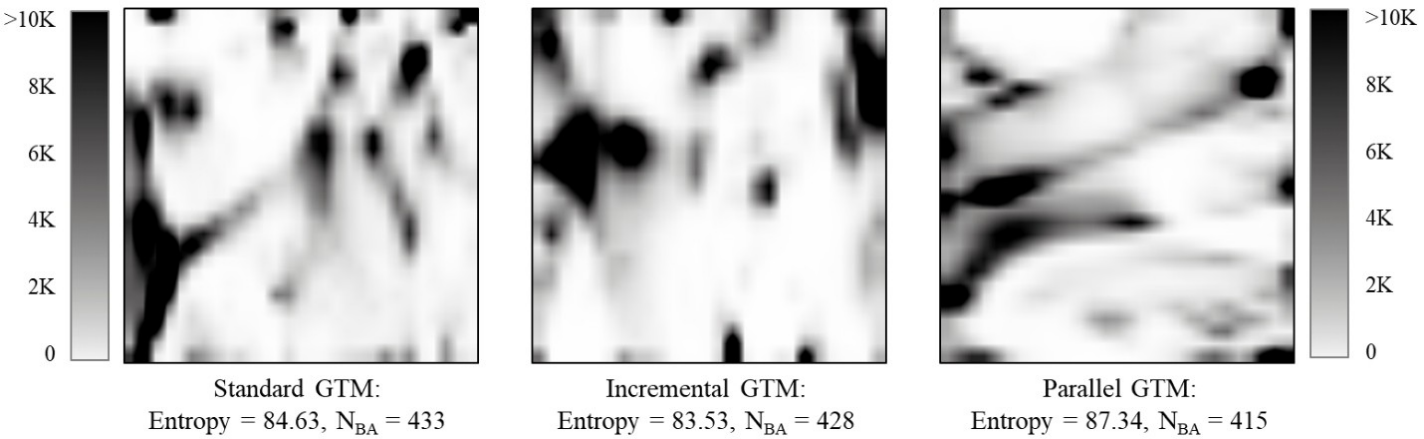
GTM density landscapes trained by sGTM, iGTM and pGTM algorithms using the FS of 20 K compounds. Here, 1.8M ChEMBL structures are projected, and each map is characterized by the normalized Shannon's entropy (Entropy) and the number of targets predicted with the <BA>≥0.7 (N_BA_).

Comparing the pGTM density landscapes with sGTM and iGTM density landscapes trained on the same FS (Figure 5), one can see that the maps are visually similar (a significant part of compounds is on the left half of the map). The entropy of the projections is nearly the same (85.17±1.96) for all three methods, but pGTM performs worse than two other algorithms. The best performance of the standard algorithm, sGTM, can be explained by the fact that it does not use any heuristics and approximations necessary for working with large amounts of data. In the case of the iGTM, the non‐complete convergence of this iterative algorithm could deteriorate the quality of the manifold, which, in turn, decreases the predictive performance of the corresponding classification models (N_BA_=428). As for the pGTM algorithm, its poorer performance could be a consequence of the heuristic character of the matrix averaging in the process of merging intermediate manifolds (N_BA_=415). On the other hand, pGTM trains the manifold 5–6 times faster than sGTM and iGTM (e. g., 30 minutes for pGTM to treat 20 K structures in contrast with 3 hours for sGTM on a machine with 8 CPUs and 32Gb RAM). In our opinion, this is a big advantage which makes the pGTM method more attractive, despite the slight decrease in predictive performance. However, it should be noticed that this speed‐up is due to the use of more CPU cores, i. e. gain in physical time – not necessarily a net gain in computational cost.

**Figure 6:**
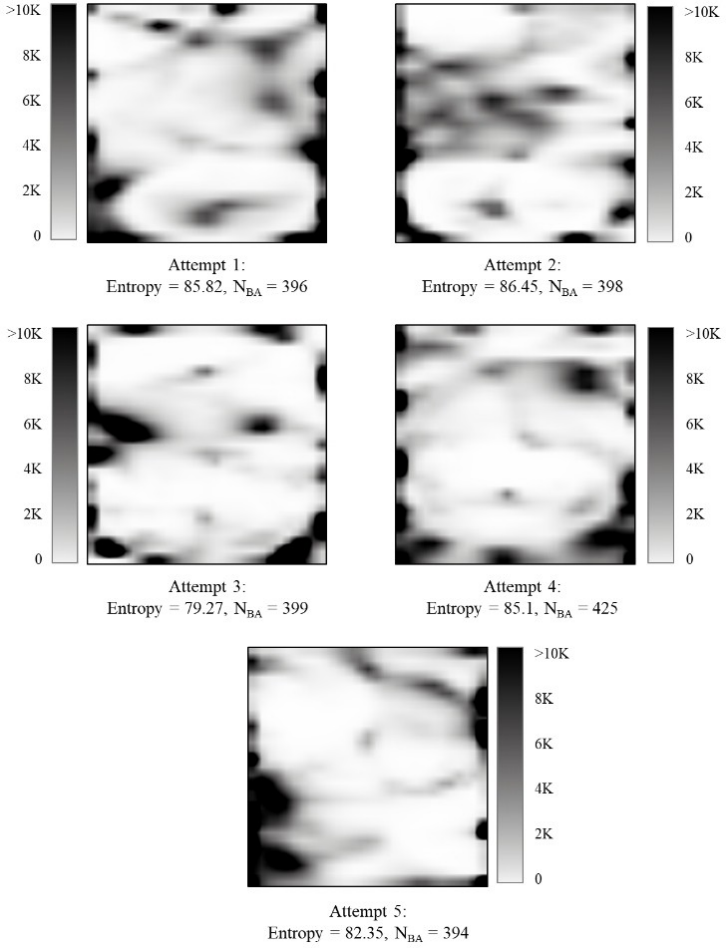
Density landscapes for the manifolds trained by pGTM on 1.8 M compounds (entire ChEMBL collection). Here, each map was trained on a reshuffled copy of the ChEMBL database and characterized by the normalized Shannon's entropy (Entropy) and the number of targets predicted with the <BA>≥0.7 (N_BA_).

In addition, it is less dependent on the order of compounds in the FS as iGTM. Indeed, the density landscapes built on five reshuffled copies of the ChEMBL database are very similar and their performance characteristics (Entropy and N_BA_) are also rather close to each other (Figure 6). Finally, pGTM can be used to treat FSs containing millions of compounds which is impossible with sGTM and hardly achievable with iGTM algorithms.

### 4.2 How Large Does a Frame Set Need to Be

**Figure 7:**
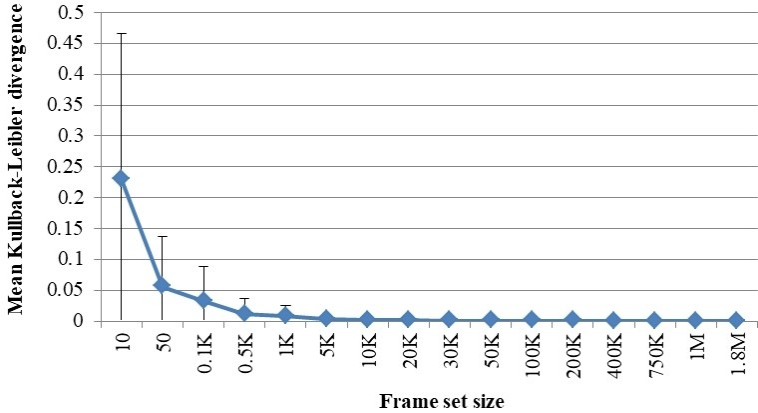
Mean Kullback‐Leibler divergences computed in *N* dimensions for pairs FS‐ChEMBL as a function of the FS size, where *N* ranges within [180; 540] (number of descriptors) depending on the FS size.

To investigate the optimal size of the FS suitable to map the entire ChEMBL collection, 80 FSs of different sizes were prepared. First, the FS representativity was compared using the Kullback‐Leibler Divergence (KLD, eq. 11). The mean and standard deviation values averaged on five repetitions are in Figure 7.

It is seen from the figure that the FS of 10 compounds is not able to describe 1.8 M molecules (KLD=0.23±0.23). However, the divergence becomes very low already for the FS of 1 K compounds, and it is 0.002 for the FS of 5 K molecules which means that 5 K already describes the ChEMBL collection very well in the current descriptor space.

**Figure 8:**
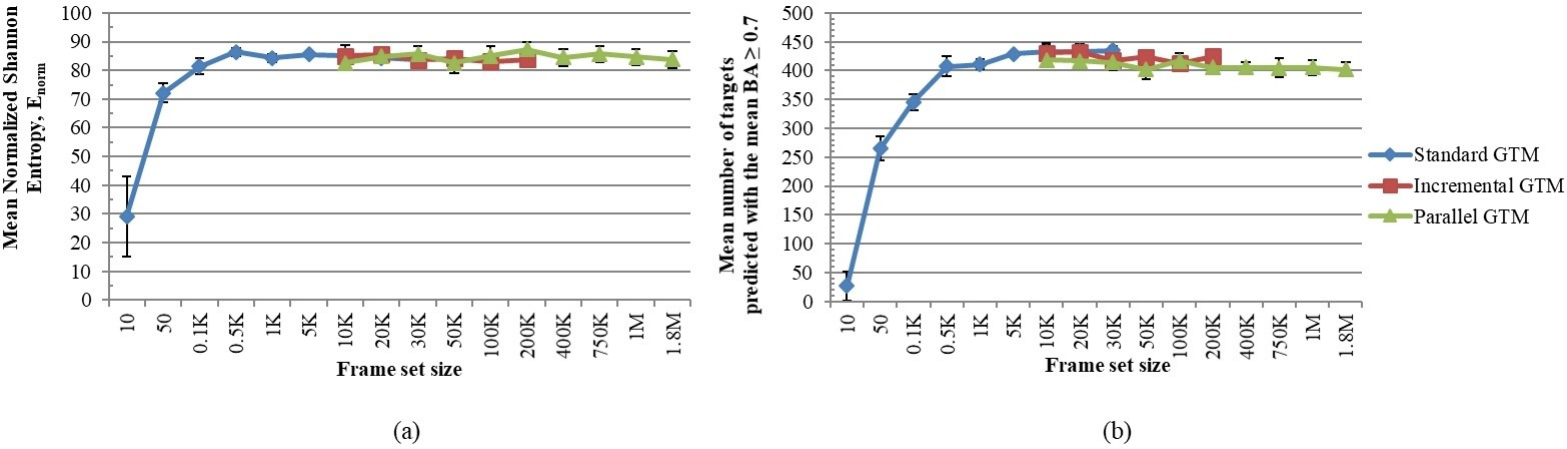
Benchmarking results: (a) the mean normalized Shannon entropy which shows the uniformity of the data distribution; (b) the mean number of targets predicted with the mean BA≥0.7.

Then, the GTMs were trained and compared in terms of the Shannon entropy (Figure 8a). It can be seen from the chart that a FS of 10 molecules does not properly cover the relevant chemical space, with the consequence that most compounds are “dumped” onto a single spot on the map. The low Shannon entropy (29±14 %) is illustrative of this fact. With the increase of the size of the FS, the manifold achieves a better expansion through relevant chemical space, which leads to more uniform data distribution over the map. The level of 86±3 % of E_norm_ (eq. 13) is reached already with the FS containing 500 compounds and it does not change significantly for the larger FSs.

Analyzing the predictive performance of the maps (Figure 8b), we have found that the plateau of the <BA> can already be reached with 5 K compounds and it remains the same for the sGTM approach. The iGTMs and pGTMs are characterized by almost the same number of targets with the <BA>≥0.7 as the standard GTMs. However, there is a slight decrease as a function of the FS size (for the parallel GTM, from 418 targets for the FSs of 10 K compounds down to 402 targets for the FSs of 1.8 M compounds). This means that we can achieve the best predictive performance with just 5000 compounds which is 0.003 % of the entire database.

It is also noteworthy that the maps trained on 500 compounds can already provide good predictive performance. This gives an opportunity to greatly accelerate the construction of GTMs for large data sets: hyperparameter tuning may be run with rather small FSs, while the final manifold at so‐far best‐found combinations of hyperparameters could be rebuilt on a larger FS if needed.

**Figure 9:**
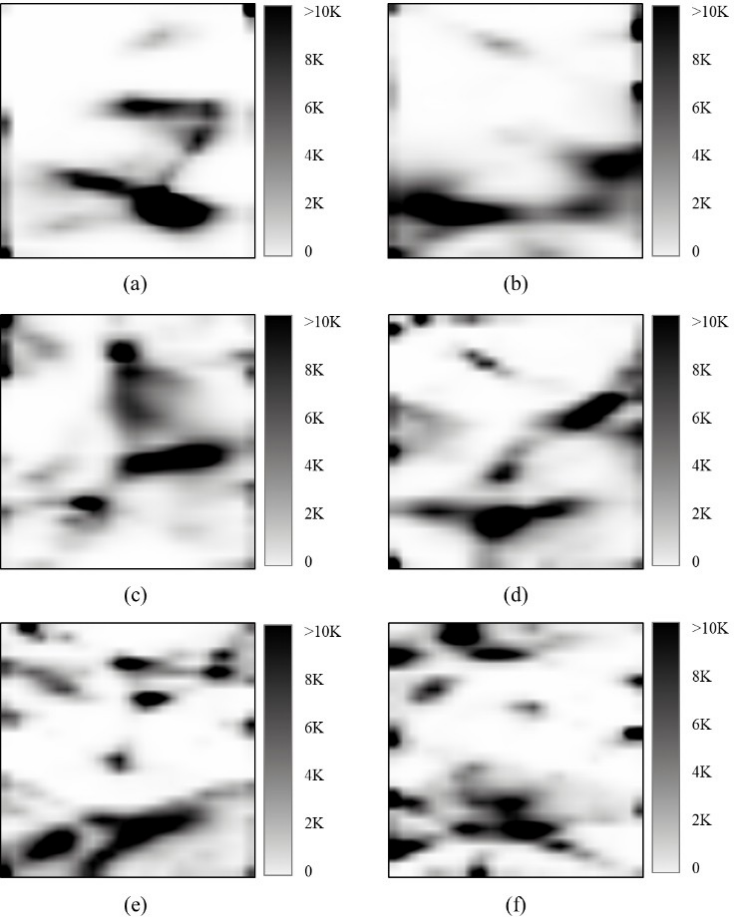
Generative Topographic Maps for the ChEMBL database built on Frame Sets of (a) 10, (b) 100, (c) 500, (d) 1000, (e) 5000, and (f) 10000 compounds using sGTM technique. The color code reflects the data density.

In terms of data visualization, the GTM density landscapes obtained with the standard approach (sGTM) support the conclusion made earlier: 5 K compounds are already enough to model the entire ChEMBL collection. On the density landscapes (Figures 9a–9f), the maximal density systematically decreases from 70 K structures down to 20 K structures and then keeps in this range. In addition, the data becomes more spread. For instance, about 7–10 clusters with a cumulated density above 10 K structures can be found for the map trained on the FS of 5 K molecules (Figure 9e) in contrast to two huge clusters shown on the map which was trained just with 10 molecules (Figure 9a). Further increasing of the FS size is not needed in this case since it does not bring any new information (Figures 9f). However, large FSs might be needed in case of huge and/or very diverse chemical collections such as CAS or Zinc where millions and billions of compounds are stored. In this case, sGTM and iGTM cannot be applied, and, therefore, pGTM can be used instead.

## 5 Conclusions

The new Parallel Generative Topographic Mapping (pGTM) algorithm was proposed. It was shown that pGTM may in principle use any, arbitrarily large FS, as it supports dispatching of the manifold fitting procedure to an arbitrary number of CPU cores or independent nodes of a cluster. Despite the slightly poorer predictive performance, pGTM is intrinsically faster (depending on the number of available CPUs) and it allows treating Frame Sets (FS) containing millions of compounds.

The method was applied to compare FSs of different sizes (10 to 1.8 M compounds) in terms of their representativity, and the trained maps were compared in terms of uniformity of data distribution, predictive performance and data visualization. It was shown that FSs with 500 compounds already produce the map of enough quality, whereas the maps with the best predictive performance (in terms of Balanced Accuracy) can be obtained with 5,000 compounds (approximately 440 targets were predicted with the mean BA≥0.7). Considering the fact that 0.003 % (5,000 structures) of the chemical collection is already enough to describe (in the framework of the GTM approach) the ChEMBL database of 1.8 M compounds, we can assume that this might be the case as well for larger chemical databases containing millions of synthesized and billions of computer‐generated structures. The study suggests that relevant mapping of the billion‐sized libraries should by no means require frame sets above a million of compounds, Frame Sets which can be handled, as shown, by the pGTM algorithm.

## Abbreviations

GTM: Generative Topographic Mapping
LLh: Log‐Likelihood
FS: Frame Set
RBF: Radial Basis Function
PCA: Principal Component Analysis
BA: Balanced accuracy
AD: Applicability Domain

## Conflict of Interest

None declared.
